# Trends in postpartum hemorrhage from 2000 to 2009: a population-based study

**DOI:** 10.1186/1471-2393-12-108

**Published:** 2012-10-11

**Authors:** Azar Mehrabadi, Jennifer A Hutcheon, Lily Lee, Robert M Liston, KS Joseph

**Affiliations:** 1 Department of Obstetrics and Gynaecology, University of British Columbia and the Children’s and Women’s Health Centre of British Columbia, Room E418B, Shaughnessy Bldg, 4500 Oak Street, Vancouver, BC V6H 3N1, Canada; 2 School of Population and Public Health, University of British Columbia, Vancouver, BC, Canada; 3 Perinatal Services BC, Vancouver, BC, Canada

## Abstract

**Background:**

Postpartum hemorrhage, a major cause of maternal death and severe maternal morbidity, increased in frequency in Canada between 1991 and 2004. We carried out a study to describe the epidemiology of postpartum hemorrhage in British Columbia, Canada, between 2000 and 2009.

**Methods:**

The study population included all women residents of British Columbia who delivered between 2000 and 2009. Data on postpartum hemorrhage by subtypes and severity were obtained from the British Columbia Perinatal Data Registry. Among women with postpartum hemorrhage, severe cases were identified by the use of blood transfusions or procedures to control bleeding. Rates of postpartum hemorrhage and changes over time were assessed using rates, rate ratios and 95% confidence intervals (CI).

**Results:**

The rate of postpartum hemorrhage increased by 27% (95% CI 21-34%) between 2000 and 2009 (from 6.3% to 8.0%), while atonic postpartum hemorrhage rates increased by 33% (95% CI 26-41%) from 4.8% to 6.4%. Atonic postpartum hemorrhage with blood transfusion increased from 17.8 to 25.5 per 10,000 deliveries from 2000 to 2009 and atonic postpartum hemorrhage with either suturing of the uterus, ligation of pelvic vessels or embolization increased from 1.8 to 5.6 per 10,000 deliveries from 2001 to 2009. The increase in atonic postpartum hemorrhage was most evident between 2006 and 2009 and occurred across regions, hospitals and various maternal, fetal and obstetric characteristics.

**Conclusions:**

Atonic postpartum hemorrhage and severe atonic postpartum hemorrhage increased in British Columbia between 2000 and 2009. Further research is required to identify the cause of the increase.

## Background

Postpartum hemorrhage is a major cause of maternal death worldwide and an important cause of severe maternal morbidity in high income countries [1]. In its severe form, postpartum hemorrhage represents a life-threatening obstetrical emergency [2]. Postpartum hemorrhage rates have increased in Canada and elsewhere from 1991 to 2004 [3]. Initial reports from Canada identified the problem as a rise in rates of severe postpartum hemorrhage, specifically an increase in rates of postpartum hemorrhage requiring hysterectomy between 1991 and 1999 [4]. This was followed by reports which showed that the epidemic was restricted to one type of postpartum hemorrhage, namely, atonic postpartum hemorrhage [5]. Increases in postpartum hemorrhage and severe postpartum hemorrhage have also been reported in Australia, Ireland, Scotland, Norway and the United States during the same time period [6-9]. The increase in postpartum hemorrhage has occurred against a background of increases in older maternal age, obesity, multiple births, deliveries to women with a previous cesarean, induction and augmentation of labour, and cesarean deliveries [5,6,8]. However, the causes for the increase in atonic postpartum hemorrhage remain unclear [5,10,11]. We carried out a study to characterize continuing trends in postpartum hemorrhage beyond 2004, with the goal of identifying potential causative factors and the clinical and population health implications. Our objective was to estimate the incidence of overall postpartum hemorrhage and its subtypes in British Columbia, Canada, between 2000 and 2009 and to describe the epidemiologic features of postpartum hemorrhage.

## Methods

The study population included all women, residents of British Columbia, who delivered between 2000 and 2009. Data were obtained from the British Columbia Perinatal Data Registry, a population-based registry whose purpose is to collect and disseminate perinatal data for research, surveillance and planning. The database contains information on approximately 99% of births in the province, including detailed clinical and diagnostic information. Standardized forms were filled-out by care providers (physicians, midwives, obstetricians, and nurses) during pregnancy, labour and delivery and postpartum, and then compiled and coded by trained data abstractors. Accuracy and validity of data is ensured through ongoing quality checks by staff, automated data rules and ongoing use in research and surveillance. All women who gave birth between April 1^st^, 2000 and March 31^st^, 2010 (hereafter referred to as years 2000 to 2009) and who resided in British Columbia were included in the study. This study received ethical approval from the University of British Columbia Children’s & Women’s Health Centre Research Ethics Board.

Postpartum hemorrhage in Canada is defined as an estimated blood loss of ≥500 mL after vaginal delivery or ≥1000 mL after cesarean delivery or as otherwise diagnosed by a care provider. Postpartum hemorrhage diagnoses were based on International Classification of Diseases (ICD) codes recorded in the Perinatal Database (ICD-9 from 2000 to 2003 and ICD-10 from 2004 to 2009). Subtypes of postpartum hemorrhage identified with ICD-9 or ICD-10 diagnostic codes included: 1) postpartum hemorrhage due to retained placenta (third stage hemorrhage), 2) postpartum hemorrhage due to uterine atony (occurring within 24 hours following delivery), 3) delayed and secondary postpartum hemorrhage (occurring after the first 24 hours following delivery) and 4) postpartum hemorrhage due to coagulation defects. Code details for postpartum hemorrhage did not change between ICD-9 and ICD-10.

Severe cases were defined as postpartum hemorrhage diagnosis in conjunction with blood transfusion, hysterectomy, or other procedures to control bleeding. Blood transfusion was defined as receipt of one or more units of whole or packed red blood cells. Hysterectomy and procedures to control bleeding were based on the Canadian Classification of Health Interventions (CCI), which was used from April 1^st^, 2001 onwards. Interventions and procedures to control bleeding in conjunction with a diagnosis of postpartum hemorrhage included hysterectomy, bimanual uterine compression and massage, uterine (and vaginal) packing, ligation of pelvic vessels, and embolization of pelvic vessels (see Additional file 1: Table S1 for all diagnostic codes and procedures). Between 2001 and 2003, 2,156 of 118,987 women (1.8%) were missing CCI procedure codes for hysterectomy or procedures to control bleeding and so equivalent Canadian Classification of Diagnostic, Therapeutic and Surgical Procedures (CCP) codes for hysterectomy were used (Additional file 1: Table S1). For other procedures to control bleeding, CCP codes could not be used as these procedure codes were introduced in the CCI. However exclusion of records made very little difference to either rates or statistical test results, so patients were included in the reported results.

Maternal, fetal and obstetrical characteristics were identified according to fields recorded in the Perinatal Data Registry. Maternal characteristics included parity, age, and body mass index (BMI) and fetal characteristics included plurality, birth weight and gestational age. Obstetrical factors included spontaneous or instrumental vaginal delivery (with vacuum or forceps), cesarean delivery with or without labour, previous cesarean delivery, any method of labour induction, augmentation with oxytocin, and administration of epidural analgesia. Geographic regions of British Columbia were defined by the regional health authority of the mother’s residence, and hospital volume was defined by number of deliveries per year, in the year of delivery. Length of hospital stay greater than 4 days and hospital stay greater than 7 days were used as a measure of the burden of illness associated with postpartum hemorrhage. The 4 day cut-off was chosen as it is greater than the average stay for both cesarean and vaginal deliveries, while the 7 days postpartum cut-off has previously been used as an indicator for severe maternal morbidity [12,13].

Rates and exact binomial 95% confidence intervals were estimated for overall postpartum hemorrhage (and its subtypes), severe postpartum hemorrhage, and severe atonic postpartum hemorrhage for the period 2000 to 2009 and for each year within this time period. Temporal trends were assessed both by contrasting the rates in 2000 or 2001 with rates in 2009 using rate ratios (RR) and 95% confidence limits (95% CI). Temporal trends were also examined across all the years in the study using a chi-square test for linear trends in proportions. Statistical significance of differences was assessed based on two-sided p values and a p value <0.05 was considered statistically significant. STATA version 11.2 was used to model smoothed rates and 95% confidence intervals overlaid on observed semi-annual rates [14]. Calendar time was modelled using a restricted cubic spline, thereby avoiding linearity assumptions and loss of information from categorizing the continuous time variable [15]. For all other analysis, we used SAS version 9.2 statistical software and Epi Info ( http://wwwn.cdc.gov/epiinfo/).

## Results

**Figure 1:**
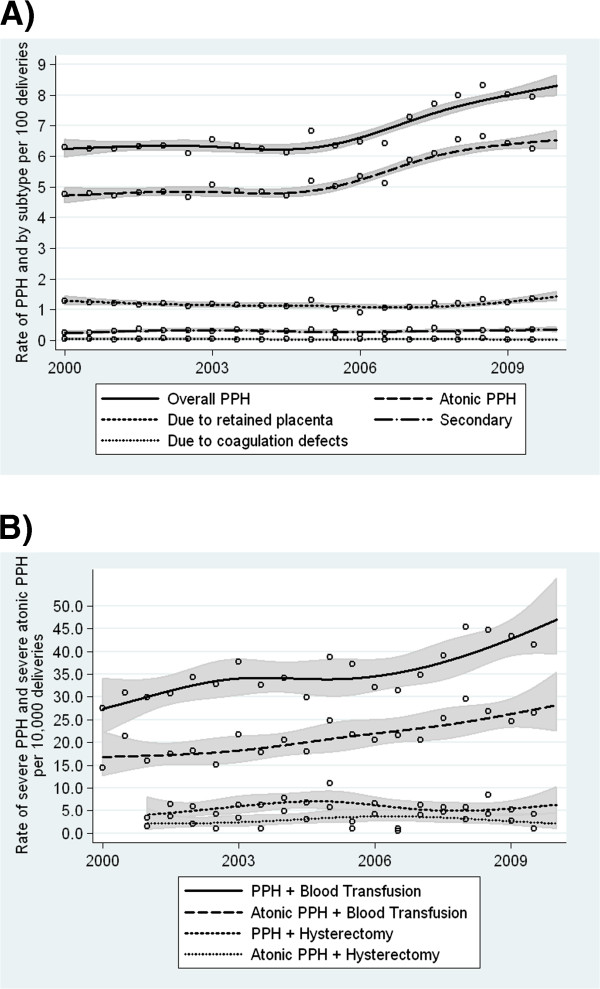
**Temporal trends in postpartum hemorrhage (PPH) by subtype (Figure**1A**), and severe PPH and severe atonic PPH (Figure**1B**).** Smoothed rates with 95% confidence interval are overlaid on observed semi-annual rates.

Between 2000 and 2009, 412,093 women residents of British Columbia delivered either a live birth or stillbirth and were included in the study. Postpartum hemorrhage rates increased from 6.3% in 2000 to 8.0% in 2009, a 27% increase (95% CI 21-34%), while atonic postpartum hemorrhage increased from 4.8% in 2000 to 6.4% in 2009, a 33% increase (95% CI 26-41%, Table 1). The increase in postpartum hemorrhage and atonic postpartum hemorrhage was particularly evident between 2006 and 2009 (Figure 1A). Overall postpartum hemorrhage and atonic postpartum hemorrhage showed an increasing linear trend between 2000 and 2009 (p<0.001). Other subtypes of postpartum hemorrhage did not show similar increasing trends (Figure 1A); there was a 40% (95% CI 9-81%) increase in secondary postpartum hemorrhage from 0.25% in 2000 to 0.35% in 2009, but the temporal trend using information for all years was not significant (p=0.27, Table 1).

**Table 1:** Temporal trends in postpartum hemorrhage (PPH), PPH subtypes, severe PPH and severe atonic PPH in British Columbia, 2000-2009

	**All years**	**PPH rate**				**2009 vs. 2000/2001**		**P for trend**^2^
	**n**^1^	**2000**	**2001**	**2008**	**2009**	**RR**	**95% CI**	
**PPH (rates per 100 deliveries):**								
All PPH	28221	6.3	6.3	8.2	8.0	1.27	1.21 - 1.34	<0.001
Due to retained placenta	4859	1.3	1.2	1.3	1.3	1.02	0.90 - 1.15	0.81
Atonic	22100	4.8	4.8	6.6	6.3	1.33	1.26 - 1.41	<0.001
Secondary	1276	0.25	0.34	0.29	0.35	1.40	1.09 - 1.81	0.27
Due to coagulation defects	178	0.05	0.05	0.05	0.03	0.51	0.25 - 1.05	0.14
**Severe PPH (rates per 10,000 deliveries):**								
PPH + blood transfusion	1468	29.1	30.3	45.1	42.5	1.46	1.16 - 1.84	<0.001
PPH + hysterectomy	215	-	4.8	7.1	4.7	0.99	0.53 - 1.84	0.76
PPH + suturing of uterus	65	-	0	3.4	3.2	-	-	<0.001
PPH + bimanual compression and massage	2255	-	27.7	98.1	95.6	3.45	2.79 - 4.25	<0.001
PPH + uterine (and vaginal) packing	223	-	4.0	6.4	8.1	2.02	1.12 - 3.63	0.02
PPH + ligation of pelvic vessels	81	-	1.3	3.9	4.5	3.58	1.34 - 9.55	<0.001
PPH + embolization of pelvic vessels	60	-	0.50	2.5	1.4	2.69	0.54 - 13.32	0.01
PPH + Suture, ligation, or embolization	186	-	1.8	9.1	7.0	3.97	1.75 - 9.01	<0.001
**Severe atonic PPH (rates per 10,000 deliveries)**								
Atonic PPH + blood transfusion	876	17.8	16.6	28.2	25.5	1.43	1.07 - 1.93	<0.001
Atonic PPH + hysterectomy	108	-	2.5	3.6	1.8	0.72	0.28 - 1.81	0.37
Atonic PPH + suturing of uterus	55	-	0	2.7	2.9	-	-	<0.001
Atonic PPH + bimanual compression and massage	1964	-	23.5	85.3	81.8	3.49	2.78 - 4.38	<0.001
Atonic PPH + uterine (and vaginal) packing	174	-	3.0	5.9	5.9	1.93	0.97 - 3.82	0.02
Atonic PPH + ligation of pelvic vessels	68	-	1.3	3.9	3.6	2.87	1.05 - 7.83	<0.001
Atonic PPH + embolization of pelvic vessels	43	-	0.50	2.0	1.1	2.24	0.43 - 11.55	0.01
Atonic PPH + Suture, ligation, or embolization	150	-	1.8	8.0	5.6	3.20	1.38 - 7.40	<0.001

^1^n refers to number of cases of the outcome of interest.

^2^P value based on data for all years between 2000 (or 2001) and 2009.

RR denotes rate ratio and 95% CI denotes 95% confidence intervals.

Severe postpartum hemorrhage increased significantly according to every indicator analyzed except for postpartum hemorrhage with hysterectomy. Postpartum hemorrhage with blood transfusion increased from 29.1 to 42.5 per 10,000 deliveries from 2000 to 2009 (an increase of 46%, 95% CI 16-84%), while postpartum hemorrhage with uterine (and vaginal) packing increased by 102% (95% CI 12-263%) from 2001 to 2009. Postpartum hemorrhage with either suturing of the uterus, ligation of pelvic vessels or embolization increased by 297% (95% CI 75-801%, Table 1). Severe atonic postpartum hemorrhage demonstrated a trend similar to trends in severe overall postpartum hemorrhage; all procedures indicating severe atonic postpartum hemorrhage increased significantly over time (Table 1). Atonic postpartum hemorrhage with blood transfusion increased from 17.8 per 10,000 deliveries in 2000 to 25.5 per 10,000 deliveries in 2009 (43% increase, 95% CI 7-93%), while atonic postpartum hemorrhage with either suturing of the uterus, ligation of pelvic vessels or embolization increased from 1.8 in 2001 to 5.6 per 10,000 deliveries in 2009 (220% increase, 95% CI 38-640%). Most of the observed increase in overall postpartum hemorrhage with blood transfusion occurred between 2006 and 2009, while atonic postpartum hemorrhage with blood transfusion rates increased at a constant rate between 2003 and 2009 (Figure 1B).

Atonic postpartum hemorrhage increased significantly between 2000 and 2009 across all regions (except for the Northern Health Authority which showed a decrease) and increased significantly across hospitals irrespective of hospital volume (except for those with 1000–1499 deliveries per year, Table 2). Atonic postpartum hemorrhage rates were higher among the large volume hospitals as compared with the small volume hospitals (6.1% compared with 4.5%). Supplementary analysis showed that women residing in the Northern Health Authority were younger, more likely to be multiparous, and less likely to receive epidural analgesia or undergo an instrumental vaginal delivery. Rates of epidural analgesia also decreased slightly (p<0.05) in this region, while rates increased significantly in all other regions.

**Table 2:** Atonic postpartum hemorrhage by region and hospital volume, British Columbia, 2000-2009

**Region (by health authority of mother's residence)**	**All years**		**Rate per 100 deliveries by year**				**2009 vs. 2000**		**P for trend**^**2**^
	**n**^**1**^	**rate**	**2000**	**2001**	**2008**	**2009**	**RR**	**95% CI**	
Fraser	8340	5.2	5.0	4.9	6.6	6.0	1.19	1.09-1.30	<0.001
Interior	2769	4.7	3.9	3.4	5.8	7.0	1.81	1.54-2.11	<0.001
Northern	1694	4.9	5.2	4.8	5.6	4.1	0.79	0.64-0.97	0.03
Vancouver Coastal	6995	7.1	5.4	6.0	8.8	8.3	1.55	1.39-1.72	<0.001
Vancouver Island	2206	3.7	3.8	3.8	4.4	4.9	1.29	1.09-1.53	<0.001
**Hospital Volume (by deliveries per year)**									
<500	2537	4.5	5.0	4.1	5.6	5.2	1.04	0.88-1.2	<0.001
500-999	2363	4.6	3.8	4.0	5.6	5.6	1.49	1.27-1.75	<0.001
1000-1499	3369	4.2	5.6	4.4	5.3	4.0	0.71	0.61-0.84	0.88
1500-2499	3851	6.3	1.5	4.9	8.4	8.2	5.45	4.30-6.92	<0.001
≥2500	9980	6.1	5.6	5.5	6.8	6.5	1.15	1.05-1.25	<0.001

^1^n refers to number of cases of atonic postpartum hemorrhage.

^2^P value based on data for all years between 2000 and 2009.

RR denotes rate ratio and 95% CI denotes 95% confidence intervals.

Atonic postpartum hemorrhage increased significantly between 2000 and 2009 across all maternal, fetal and obstetric characteristics under study with the exception of birthweight <2500 grams, and gestational ages <28 weeks or ≥42 weeks (Table 3). An increase in atonic postpartum hemorrhage was observed both among women with vaginal deliveries and cesarean deliveries, and regardless of whether a women had a previous cesarean delivery. Among cesarean deliveries, atonic postpartum hemorrhage increased significantly among all cesarean sub-categories, including cesareans without labour, those with spontaneous labour and those who had labour induction. Among women with a vaginal delivery, atonic postpartum hemorrhage increased more markedly among those with an instrumental vaginal delivery. Atonic postpartum hemorrhage increased significantly regardless of whether a woman had her labour induced, was augmented with oxytocin, or received epidural analgesia. The percent increase, however, was higher among women receiving these procedures.

**Table 3:** Temporal Trends in atonic postpartum hemorrhage by maternal, fetal and obstetrical characteristics, British Columbia, 2000-2009

	**All years**		**Rate per 100 deliveries**				**2009 vs. 2000**		**P for trend**^**2**^
	**n**^**1**^	**rate**	**2000**	**2001**	**2008**	**2009**	**RR**	**95% CI**	
**Parity**									
Nulliparous	12,237	6.5	5.5	5.5	8.1	7.6	1.39	1.29-1.51	<0.001
Parous	9,863	4.4	4.2	4.2	5.3	5.2	1.24	1.14-1.35	<0.001
**Maternal age (years)**									
<20	953	6.2	6.1	5.0	8.5	7.6	1.26	0.97-1.62	<0.001
20-34	16,641	5.4	4.8	4.8	6.6	6.4	1.34	1.26-1.43	<0.001
≥35	4,506	5.2	4.4	4.7	6.3	5.9	1.34	1.18-1.53	<0.001
**BMI**									
Underweight	1,032	6.0	4.8	5.2	7.4	6.8	1.43	1.09-1.87	<0.001
Normal	9,960	5.7	5.1	5.1	6.9	6.8	1.33	1.22-1.47	<0.001
Overweight	2,977	5.1	4.5	4.5	6.2	6.2	1.39	1.19-1.62	<0.001
Obese	1,583	4.9	4.2	4.4	6.4	5.5	1.31	1.06-1.63	<0.001
**Plurality**									
Singleton	21,529	5.3	4.7	4.7	6.5	6.3	1.33	1.25-1.41	<0.001
Multiple	571	9.2	7.1	10.8	11.3	9.4	1.33	0.91-1.95	<0.001
**Birthweight (grams)**									
<2500	794	3.7	3.2	4.0	5.0	3.7	1.17	0.85-1.61	0.03
2500-3999	17,353	5.2	4.5	4.5	6.3	6.2	1.37	1.29-1.47	<0.001
≥4000	3,939	7.2	6.6	6.4	9.1	8.2	1.24	1.09-1.41	<0.001
**Gestational age (weeks)**									
<28	68	2.0	2.14	0.69	3.13	1.5	0.71	0.25-2.01	0.53
28-36	1,477	4.5	3.3	4.2	6.0	5.2	1.55	1.21-1.97	<0.001
37-41	20,115	5.5	4.9	4.8	6.7	6.5	1.33	1.25-1.41	<0.001
≥42	396	6.2	5.8	5.3	6.9	5.9	1.03	0.68-1.57	0.52
**Vaginal delivery**									
Spontaneous	14,400	5.8	5.1	5.2	7.1	6.8	1.33	1.24-1.42	<0.001
Instrumental	5,141	11.5	8.9	10.3	14.6	14.2	1.59	1.42-1.79	<0.001
**Cesarean delivery**									
No labour	912	1.7	1.6	1.3	2.1	2.1	1.28	0.94-1.73	<0.001
With labour, no induction	1,001	2.4	1.6	1.5	3.5	3.0	1.92	1.43-2.58	<0.001
With induction	646	2.9	1.9	1.5	3.9	3.5	1.74	1.20-2.54	<0.001
**Previous cesarean**									
Yes	1,426	2.5	2.7	2.3	3.2	2.9	1.08	0.86-1.34	0.001
No	20,674	5.8	5.1	5.1	7.2	6.9	1.37	1.30-1.46	<0.001
**Induction of labour**									
Yes	5,648	6.4	5.4	5.4	8.4	7.7	1.41	1.26-1.58	<0.001
No	16,452	5.1	4.6	4.6	6.1	6.0	1.31	1.22-1.40	<0.001
**Augmentation of labour (oxytocin)**									
Yes	4,561	7.2	5.7	6.1	8.9	8.1	1.41	1.24-1.61	<0.001
No	17,539	5.0	4.6	4.5	6.1	6.0	1.31	1.23-1.39	<0.001
**Epidural analgesia**									
Yes	8,096	7.0	5.4	6.0	9.0	7.8	1.44	1.30-1.59	<0.001
No	14,004	4.7	4.6	4.3	5.6	5.8	1.27	1.19-1.36	<0.001

^1^n refers to number of cases of atonic postpartum hemorrhage.

^2^P value based on data for all years between 2000 and 2009.

RR denotes rate ratio and 95% CI denotes 95% confidence intervals.

Trends in atonic postpartum hemorrhage with blood transfusion were more variable over time across maternal, fetal and obstetric characteristics, likely due to smaller numbers for this outcome (Table 4). Significant and non-significant increases were observed across many maternal, fetal and obstetric categories. Notably, increases in atonic postpartum hemorrhage with blood transfusion were evident among both spontaneous and instrumental vaginal deliveries, although the increase was greater for instrumental vaginal births. Additionally, increases were evident among women irrespective of use of labour induction, oxytocin augmentation, or epidural analgesia. Increases in atonic postpartum hemorrhage with blood transfusion among women with a cesarean delivery were only observed among cesareans following labour induction. Although atonic postpartum hemorrhage rates were higher for vaginal as compared with cesarean deliveries (Table 3), rates of atonic postpartum hemorrhage with blood transfusion (as well as all other types of severe atonic postpartum hemorrhage – data not shown) were higher among cesarean as compared with vaginal deliveries (Table 4).

**Table 4:** Temporal Trends in atonic postpartum hemorrhage with blood transfusion by maternal, fetal and obstetrical characteristics, British Columbia, 2000-2009

**Parity**	**All years**		**Rate per 10,000 deliveries**				**2009 vs. 2000**		**P for trend**^**2**^
	**n**^**1**^	**rate**	**2000**	**2001**	**2008**	**2009**	**RR**	**95% CI**	
Nulliparous	534	15.3	20.1	19.9	39.5	31.3	1.56	1.04-2.34	<0.001
Parous	342	28.3	16.0	14.1	18.3	20.4	1.28	0.83-1.98	0.04
**Maternal Age (in years)**									
<20	56	36.6	27.3	28.6	47.7	55.8	2.05	0.67-6.24	0.07
20-34	612	19.8	15.8	15.0	27.3	25.5	1.61	1.13-2.30	<0.001
≥35	208	23.8	23.4	20.4	28.4	21.2	0.91	0.48-1.70	0.41
**BMI**									
Underweight	41	23.9	10.4	26.4	25.5	18.4	1.78	0.30-10.62	0.10
Normal	362	20.6	16.3	19.1	30.4	20.4	1.25	0.77-2.03	0.03
Overweight	115	19.8	17.3	13.6	25.0	24.1	1.39	0.62-3.08	0.02
Obese	58	17.8	10.1	6.5	27.3	18.6	1.85	0.48-7.14	0.09
**Plurality**									
Singleton	832	20.5	17.0	15.3	27.0	24.8	1.46	1.07-1.97	<0.001
Multiple	44	70.7	76.3	113.9	105.6	68.0	0.89	0.24-3.30	0.84
**Birthweight (in grams)**									
<2500	59	27.8	5.1	59.2	39.4	29.9	5.92	0.73-48.08	0.69
2500-3999	637	19.0	17.2	13.8	22.5	22.0	1.28	0.91-1.81	0.002
≥4000	177	32.3	26.0	18.8	58.3	45.4	1.75	0.92-3.32	<0.001
**Gestational age (in weeks)**									
<28	7	20.1	0.0	34.5	24.0	21.8	-	-	0.69
28-36	110	33.8	25.1	36.6	45.7	46.4	1.85	0.77-4.45	0.05
37-41	739	20.0	17.1	15.3	27.0	23.3	1.37	0.99-1.89	<0.001
≥42	17	26.6	35.2	0.0	16.5	34.0	0.96	0.16-5.75	0.61
**Vaginal delivery**									
Spontaneous	321	12.9	11.8	10.2	17.6	16.4	1.39	0.87-2.21	0.004
Instrumental	244	54.7	31.2	41.4	73.3	73.6	2.36	1.29-4.32	<0.001
**Cesarean delivery**									
No labour	110	20.7	26.9	18.1	21.5	24.7	0.92	0.41-2.04	0.57
With labour, no induction	129	30.4	31.2	30.0	33.7	29.0	0.93	0.43-2.01	0.59
With induction	72	32.2	20.3	9.3	65.0	28.8	1.42	0.42-4.83	0.01
**Previous cesarean**									
Yes	111	19.8	29.5	18.4	27.6	29.1	0.99	0.50-1.97	0.60
No	765	21.5	16.2	16.4	28.3	24.9	1.53	1.10-2.13	<0.001
**Induction of labour**									
Yes	236	26.9	19.9	18.9	44.3	31.3	1.57	0.86-2.86	0.002
No	640	19.7	17.3	16.0	24.1	24.0	1.39	0.99-1.96	<0.001
**Augmentation of labour (oxytocin)**									
Yes	172	27.3	23.2	15.4	38.4	38.0	1.64	0.86-3.12	<0.001
No	704	20.2	16.9	16.9	26.2	23.1	1.37	0.98-1.92	<0.001
**Epidural analgesia**									
Yes	340	29.4	23.4	20.9	40.2	35.4	1.51	0.92-2.48	<0.001
No	536	18.1	15.9	15.1	23.2	21.4	1.35	0.93-1.96	0.003

^1^n refers to number of cases of atonic postpartum hemorrhage with blood transfusion.

^2^P value based on data for all years between 2000 and 2009.

RR denotes rate ratio and 95% CI denotes 95% confidence intervals.

Women with postpartum hemorrhage were more likely to have long hospital stays; 9.5% of women with postpartum hemorrhage had a length of stay greater than 4 days compared with 7.2% of women without postpartum hemorrhage (RR=1.32, 95% CI 1.27-1.37), and 1.3% had a length of stay greater than 7 days as compared with 0.7% of women without postpartum hemorrhage (RR=1.88, 95% CI 1.69-2.10).

## Discussion

Previous studies have reported an increase in postpartum hemorrhage between 1991 and 2004 and in severe postpartum hemorrhage between 2003 and 2007 in Canada [5,16]. Our population-based study from British Columbia demonstrated that postpartum hemorrhage rates continued to increase until 2009 with a steep increase between 2006 and 2009. The increase was driven by a 33% rise in atonic postpartum hemorrhage. There was a 43% increase in atonic postpartum hemorrhage with blood transfusion and a 220% increase in atonic postpartum hemorrhage with either uterine suturing, ligation of pelvic vessels or embolization.

The increase in overall and atonic postpartum hemorrhage since 2006 coincides with a change in the definition of postpartum hemorrhage in the Canadian Institute for Health Information 2006 coding standards [17]. Increases in postpartum hemorrhage diagnoses around this time period were also reported elsewhere in Canada. A recent database report from Nova Scotia documented a 24% increase in postpartum hemorrhage from 2004 to 2005, from 5.0% to 6.2%, followed by a 29% increase between 2006 and 2007, from 6.2% to 8.0% [18]. Although these increases may be partly explained by this definition change, the increase was also observed for severe postpartum hemorrhage and severe atonic postpartum hemorrhage (as measured by concurrent blood transfusion or procedures to control bleeding). The latter are considered objective and clinically important measures of severity. Additionally, the increase did not occur for all subtypes of postpartum hemorrhage as would be expected with a change in the definition of postpartum hemorrhage.

Postpartum hemorrhage rates increased across the majority of maternal, fetal and obstetric characteristics and this finding does not point to any single cause. Notably, atonic postpartum hemorrhage increased even among cesarean deliveries with no labour or labour induction. One mechanism suggested for uterine atony (and for the current postpartum hemorrhage epidemic [10]) is desensitization of uterine tissue to oxytocin due to its liberal administration during labour induction and augmentation [19]. However the observed increase in postpartum hemorrhage occurred even among women not induced or augmented, highlighting the fact that other causes may be responsible. Factors not associated with an increasing trend in postpartum hemorrhage (such as gestational age <28 weeks) typically had a low frequency and such findings are not inconsistent with an across-the-board increase in postpartum hemorrhage. The higher rates of atonic postpartum hemorrhage among vaginal deliveries (as opposed to cesarean deliveries) may be related to differing definitions of postpartum hemorrhage for these two routes of delivery. Cesarean deliveries were associated with higher rates of severe atonic postpartum hemorrhage than vaginal deliveries (Table 4).

The declining trend in postpartum hemorrhage among women from the Northern Health Authority may provide some etiologic insight into the increases seen elsewhere. The inter-relationship between risk factors for postpartum hemorrhage such epidural analgesia, oxytocin induction and instrumental vaginal delivery require further investigation in order to delineate their relative contributions to the change in postpartum hemorrhage rates. On the other hand, the Northern Health Authority represented one of the smaller regions and rates by year fluctuated somewhat even though the linear trend was statistically significant (Table 2). The fact that postpartum hemorrhage with hysterectomy did not increase over the latter half of the study period is a welcome development as Canada [3] (and British Columbia in particular [13]) has higher postpartum hemorrhage with hysterectomy rates as compared with other countries. Increasing use of procedures other than hysterectomy to control bleeding may be responsible for the trend in hysterectomy for postpartum hemorrhage.

Key strengths of our study include use of procedure code definitions for procedures to control bleeding that did not change over time, and postpartum hemorrhage code definitions that remained unchanged between ICD-9 and ICD-10. However, as noted, there was a change in the definition of postpartum hemorrhage in 2006. Besides blood transfusions and hysterectomies, other interventions with postpartum hemorrhage have not been reported in previous studies and it is unclear to what extent they have been consistently documented over time. Limitations of our study include use of information from a large perinatal database, which could contain some coding and transcription errors. In addition, multiple comparisons may increase the probability of falsely significant findings. Other limitations include problems with the clinical diagnosis of postpartum hemorrhage since estimation of blood loss during childbirth is difficult to standardize. Our use of different measures of severe postpartum hemorrhage was important for identifying increasing trends in clinically relevant cases of postpartum hemorrhage.

## Conclusions

Atonic postpartum hemorrhage and severe atonic postpartum hemorrhage rates continued to increase in British Columbia across a range of maternal, fetal and obstetric characteristics. The 46% increase in atonic postpartum hemorrhage with blood transfusion between 2000 and 2009 is substantial. Given that these increases were similar to findings from various countries and jurisdictions employing different definitions and distinct markers of severity, further research is required to identify and address the cause or causes of the increase in atonic postpartum hemorrhage.

## Competing interests

The authors declare that they have no financial or other competing interests.

## Author’s contributions

AM and KSJ were responsible for the intellectual content of the study proposal; they developed and articulated the conceptual framework and study design. AM, KSJ and JH developed the analytic approach. AM wrote the first draft of the manuscript and analyzed the data. JAH, LL RML and KSJ made substantial contributions to the study design and study proposal drafts; they make substantial contributions to the analysis and interpretation of data and revised the article for important intellectual content. All authors have read and approved the final manuscript.

## Pre-publication history

The pre-publication history for this paper can be accessed here:

http://www.biomedcentral.com/1471-2393/12/108/prepub

## Supplementary Material

Additional file 1 **Table S1.** International Classification of Diseases (ICD-9, ICD-10), the Canadian Classification of Diagnostic, Therapeutic and Surgical Procedures (CCP), and the Canadian Classification of Interventions (CCI) diagnosis/procedure codes used. Click here for file
