# Supplementary material for: Trends in postpartum hemorrhage from 2000 to 2009: a population-based study
Source: BMC Pregnancy Childbirth. 2012 Oct 11;12:108. doi: 10.1186/1471-2393-12-108 (PMC3534600; doi:10.1186/1471-2393-12-108)
Supplement: Additional file 1 — Table S1. International Classification of Diseases (ICD-9, ICD-10), the Canadian Classification of Diagnostic, Therapeutic and Surgical Procedures (CCP), and the Canadian Classification of Interventions (CCI) diagnosis/procedure codes used. [file 1471-2393-12-108-S1.pdf]

Table 1. Temporal trends in postpartum hemorrhage (PPH), PPH subtypes, severe PPH and severe atonic PPH in British Columbia, 2000-2009.

	All years	PPH rate				2009 vs. 2000/2001		
	n[1]	2000	2001	2008	2009	RR	95% CI	P for trend[2]
**PPH (rates per 100 deliveries):**								
All PPH	28221	6.3	6.3	8.2	8.0	1.27	1.21 - 1.34	<0.001
Due to retained placenta	4859	1.3	1.2	1.3	1.3	1.02	0.90 - 1.15	0.81
Atonic	22100	4.8	4.8	6.6	6.3	1.33	1.26 - 1.41	<0.001
Secondary	1276	0.25	0.34	0.29	0.35	1.40	1.09 - 1.81	0.27
Due to coagulation defects	178	0.05	0.05	0.05	0.03	0.51	0.25 - 1.05	0.14
**Severe PPH (rates per 10,000 deliveries):**								
PPH + blood transfusion	1468	29.1	30.3	45.1	42.5	1.46	1.16 - 1.84	<0.001
PPH + hysterectomy	215	-	4.8	7.1	4.7	0.99	0.53 - 1.84	0.76
PPH + suturing of uterus	65	-	0	3.4	3.2	-	-	<0.001
PPH + bimanual compression and massage	2255	-	27.7	98.1	95.6	3.45	2.79 - 4.25	<0.001
PPH + uterine (and vaginal) packing	223	-	4.0	6.4	8.1	2.02	1.12 - 3.63	0.02
PPH + ligation of pelvic vessels	81	-	1.3	3.9	4.5	3.58	1.34 - 9.55	<0.001
PPH + embolization of pelvic vessels	60	-	0.50	2.5	1.4	2.69	0.54 - 13.32	0.01
PPH + Suture, ligation, or embolization	186	-	1.8	9.1	7.0	3.97	1.75 - 9.01	<0.001
**Severe atonic PPH (rates per 10,000 deliveries)**								
Atonic PPH + blood transfusion	876	17.8	16.6	28.2	25.5	1.43	1.07 - 1.93	<0.001
Atonic PPH + hysterectomy	108	-	2.5	3.6	1.8	0.72	0.28 - 1.81	0.37
Atonic PPH + suturing of uterus	55	-	0	2.7	2.9	-	-	<0.001
Atonic PPH + bimanual compression and massage	1964	-	23.5	85.3	81.8	3.49	2.78 - 4.38	<0.001
Atonic PPH + uterine (and vaginal) packing	174	-	3.0	5.9	5.9	1.93	0.97 - 3.82	0.02
Atonic PPH + ligation of pelvic vessels	68	-	1.3	3.9	3.6	2.87	1.05 - 7.83	<0.001
Atonic PPH + embolization of pelvic vessels	43	-	0.50	2.0	1.1	2.24	0.43 - 11.55	0.01
Atonic PPH + Suture, ligation, or embolization	150	-	1.8	8.0	5.6	3.20	1.38 - 7.40	<0.001

[1]n refers to number of cases of the outcome of interest.
[2]P value based on data for all years between 2000 (or 2001) and 2009.
RR denotes rate ratio and 95% CI denotes 95% confidence intervals.

Table 2. Atonic postpartum hemorrhage by region and hospital volume, British Columbia, 2000-2009.

Region (by health authority of mother's residence)	All years		Rate per 100 deliveries by year				2009 vs. 2000		P for trend[2]
	n[1]	rate	2000	2001	2008	2009	RR	95% CI	
Fraser	8340	5.2	5.0	4.9	6.6	6.0	1.19	1.09-1.30	<0.001
Interior	2769	4.7	3.9	3.4	5.8	7.0	1.81	1.54-2.11	<0.001
Northern	1694	4.9	5.2	4.8	5.6	4.1	0.79	0.64-0.97	0.03
Vancouver Coastal	6995	7.1	5.4	6.0	8.8	8.3	1.55	1.39-1.72	<0.001
Vancouver Island	2206	3.7	3.8	3.8	4.4	4.9	1.29	1.09-1.53	<0.001
**Hospital Volume (by deliveries per year)**									
<500	2537	4.5	5.0	4.1	5.6	5.2	1.04	0.88-1.23	<0.001
500-999	2363	4.6	3.8	4.0	5.6	5.6	1.49	1.27-1.75	<0.001
1000-1499	3369	4.2	5.6	4.4	5.3	4.0	0.71	0.61-0.84	0.88
1500-2499	3851	6.3	1.5	4.9	8.4	8.2	5.45	4.30-6.92	<0.001
≥2500	9980	6.1	5.6	5.5	6.8	6.5	1.15	1.05-1.25	<0.001

[1]n refers to number of cases of atonic postpartum hemorrhage.
[2]P value based on data for all years between 2000 and 2009.
RR denotes rate ratio and 95% CI denotes 95% confidence intervals.

Table 3. Temporal Trends in atonic postpartum hemorrhage by maternal, fetal and obstetrical characteristics, British Columbia, 2000-2009.

	All years		Rate per 100 deliveries				2009 vs. 2000		P for trend[2]
	n[1]	rate	2000	2001	2008	2009	RR	95% CI	
**Parity**									
Nulliparous	12,237	6.5	5.5	5.5	8.1	7.6	1.39	1.29-1.51	<0.001
Parous	9,863	4.4	4.2	4.2	5.3	5.2	1.24	1.14-1.35	<0.001
**Maternal age (years)**									
<20	953	6.2	6.1	5.0	8.5	7.6	1.26	0.97-1.62	<0.001
20-34	16,641	5.4	4.8	4.8	6.6	6.4	1.34	1.26-1.43	<0.001
≥35	4,506	5.2	4.4	4.7	6.3	5.9	1.34	1.18-1.53	<0.001
**BMI**									
Underweight	1,032	6.0	4.8	5.2	7.4	6.8	1.43	1.09-1.87	<0.001
Normal	9,960	5.7	5.1	5.1	6.9	6.8	1.33	1.22-1.47	<0.001
Overweight	2,977	5.1	4.5	4.5	6.2	6.2	1.39	1.19-1.62	<0.001
Obese	1,583	4.9	4.2	4.4	6.4	5.5	1.31	1.06-1.63	<0.001
**Plurality**									
Singleton	21,529	5.3	4.7	4.7	6.5	6.3	1.33	1.25-1.41	<0.001
Multiple	571	9.2	7.1	10.8	11.3	9.4	1.33	0.91-1.95	<0.001
**Birthweight (grams)**									
<2500	794	3.7	3.2	4.0	5.0	3.7	1.17	0.85-1.61	0.03
2500-3999	17,353	5.2	4.5	4.5	6.3	6.2	1.37	1.29-1.47	<0.001
≥4000	3,939	7.2	6.6	6.4	9.1	8.2	1.24	1.09-1.41	<0.001
**Gestational age (weeks)**									
<28	68	2.0	2.14	0.69	3.13	1.5	0.71	0.25-2.01	0.53
28-36	1,477	4.5	3.3	4.2	6.0	5.2	1.55	1.21-1.97	<0.001
37-41	20,115	5.5	4.9	4.8	6.7	6.5	1.33	1.25-1.41	<0.001
≥42	396	6.2	5.8	5.3	6.9	5.9	1.03	0.68-1.57	0.52
**Vaginal delivery**									
Spontaneous	14,400	5.8	5.1	5.2	7.1	6.8	1.33	1.24-1.42	<0.001
Instrumental	5,141	11.5	8.9	10.3	14.6	14.2	1.59	1.42-1.79	<0.001
**Cesarean delivery**									
No labour	912	1.7	1.6	1.3	2.1	2.1	1.28	0.94-1.73	<0.001
With labour, no induction	1,001	2.4	1.6	1.5	3.5	3.0	1.92	1.43-2.58	<0.001
With induction	646	2.9	1.9	1.5	3.9	3.5	1.74	1.20-2.54	<0.001
**Previous cesarean**									
Yes	1,426	2.5	2.7	2.3	3.2	2.9	1.08	0.86-1.34	0.001
No	20,674	5.8	5.1	5.1	7.2	6.9	1.37	1.30-1.46	<0.001
**Induction of labour**									
Yes	5,648	6.4	5.4	5.4	8.4	7.7	1.41	1.26-1.58	<0.001
No	16,452	5.1	4.6	4.6	6.1	6.0	1.31	1.22-1.40	<0.001
**Augmentation of labour (oxytocin)**									
Yes	4,561	7.2	5.7	6.1	8.9	8.1	1.41	1.24-1.61	<0.001
No	17,539	5.0	4.6	4.5	6.1	6.0	1.31	1.23-1.39	<0.001
**Epidural analgesia**									
Yes	8,096	7.0	5.4	6.0	9.0	7.8	1.44	1.30-1.59	<0.001
No	14,004	4.7	4.6	4.3	5.6	5.8	1.27	1.19-1.36	<0.001

[1]n refers to number of cases of atonic postpartum hemorrhage.
[2]P value based on data for all years between 2000 and 2009.
RR denotes rate ratio and 95% CI denotes 95% confidence intervals.

Table 4. Temporal Trends in atonic postpartum hemorrhage with blood transfusion by maternal, fetal and obstetrical characteristics, British Columbia, 2000-2009.

	All years		Rate per 10,000 deliveries				2009 vs. 2000		P for trend[2]
	n[1]	rate	2000	2001	2008	2009	RR	95% CI	
**Parity**									
Nulliparous	534	15.3	20.1	19.9	39.5	31.3	1.56	1.04-2.34	<0.001
Parous	342	28.3	16.0	14.1	18.3	20.4	1.28	0.83-1.98	0.04
**Maternal Age (in years)**									
<20	56	36.6	27.3	28.6	47.7	55.8	2.05	0.67-6.24	0.07
20-34	612	19.8	15.8	15.0	27.3	25.5	1.61	1.13-2.30	<0.001
≥35	208	23.8	23.4	20.4	28.4	21.2	0.91	0.48-1.70	0.41
**BMI**									
Underweight	41	23.9	10.4	26.4	25.5	18.4	1.78	0.30-10.62	0.10
Normal	362	20.6	16.3	19.1	30.4	20.4	1.25	0.77-2.03	0.03
Overweight	115	19.8	17.3	13.6	25.0	24.1	1.39	0.62-3.08	0.02
Obese	58	17.8	10.1	6.5	27.3	18.6	1.85	0.48-7.14	0.09
**Plurality**									
Singleton	832	20.5	17.0	15.3	27.0	24.8	1.46	1.07-1.97	<0.001
Multiple	44	70.7	76.3	113.9	105.6	68.0	0.89	0.24-3.30	0.84
**Birthweight (in grams)**									
<2500	59	27.8	5.1	59.2	39.4	29.9	5.92	0.73-48.08	0.69
2500-3999	637	19.0	17.2	13.8	22.5	22.0	1.28	0.91-1.81	0.002
≥4000	177	32.3	26.0	18.8	58.3	45.4	1.75	0.92-3.32	<0.001
**Gestational age (in weeks)**									
<28	7	20.1	0.0	34.5	24.0	21.8	-	-	0.69
28-36	110	33.8	25.1	36.6	45.7	46.4	1.85	0.77-4.45	0.05
37-41	739	20.0	17.1	15.3	27.0	23.3	1.37	0.99-1.89	<0.001
≥42	17	26.6	35.2	0.0	16.5	34.0	0.96	0.16-5.75	0.61
**Vaginal delivery**									
Spontaneous	321	12.9	11.8	10.2	17.6	16.4	1.39	0.87-2.21	0.004
Instrumental	244	54.7	31.2	41.4	73.3	73.6	2.36	1.29-4.32	<0.001
**Cesarean delivery**									
No labour	110	20.7	26.9	18.1	21.5	24.7	0.92	0.41-2.04	0.57
With labour, no induction	129	30.4	31.2	30.0	33.7	29.0	0.93	0.43-2.01	0.59
With induction	72	32.2	20.3	9.3	65.0	28.8	1.42	0.42-4.83	0.01
**Previous cesarean**									
Yes	111	19.8	29.5	18.4	27.6	29.1	0.99	0.50-1.97	0.60
No	765	21.5	16.2	16.4	28.3	24.9	1.53	1.10-2.13	<0.001
**Induction of labour**									
Yes	236	26.9	19.9	18.9	44.3	31.3	1.57	0.86-2.86	0.002
No	640	19.7	17.3	16.0	24.1	24.0	1.39	0.99-1.96	<0.001
**Augmentation of labour (oxytocin)**									
Yes	172	27.3	23.2	15.4	38.4	38.0	1.64	0.86-3.12	<0.001
No	704	20.2	16.9	16.9	26.2	23.1	1.37	0.98-1.92	<0.001
**Epidural analgesia**									
Yes	340	29.4	23.4	20.9	40.2	35.4	1.51	0.92-2.48	<0.001
No	536	18.1	15.9	15.1	23.2	21.4	1.35	0.93-1.96	0.003

[1]n refers to number of cases of atonic postpartum hemorrhage with blood transfusion.
[2]P value based on data for all years between 2000 and 2009.
RR denotes rate ratio and 95% CI denotes 95% confidence intervals.
